# Ebola-Poe: A Modern-Day Parallel of the Red Death?

**DOI:** 10.3201/eid0812.020176

**Published:** 2002-12

**Authors:** Setu K. Vora, Sundaram V. Ramanan

Plagues and pestilence have evoked fear and awe since time immemorial. Often viewed as divine retribution, these scourges are mentioned in many cultural and religious texts, including the Bible, the Koran, and the Talmud. History itself is punctuated and shaped by epidemics, whose accounts are at the center of such literary works as Boccachio’s Decameron, Daniel Defoe’s A Journal of the Plague Year, and Gabriel Garcia Marquez’ Love in the Time of Cholera. These works provide rare insight into the impact of real epidemics. Accounts of fictional epidemics, such as Albert Camus’ The Plague or Edgar Allan Poe’s The Masque of Red Death, are even more fascinating and debatable.

Poe’s most famous works are macabre tales of terror, madness, decay, and death. The author’s life has been the subject of numerous medical and psychiatric analyses, and the effects of alcoholism (1) and seizure disorder (2) on his creativity have been studied. Review of The Fall of the House of Usher has implicated porphyria (3) for the psychopathology of Roderick Usher and his sister Madeline. But to our knowledge, a detailed medical analysis of The Masque of Red Death has not been conducted.

The tale opens with the description of a mysterious epidemic. “The ‘Red Death’ had long devastated the country. No pestilence had ever been so fatal, or so hideous. Blood was its avatar and its seal—the redness and the horror of blood. There were sharp pains, and sudden dizziness, and then profuse bleeding at the pores, with dissolution. The scarlet stains upon the body and especially upon the face of the victim, were the pest ban which shut him out from the aid and from the sympathy of his fellow-men. And the whole seizure, progress and termination of the disease, were the incidents of half an hour.”

To escape death, Prince Prospero secludes himself and a thousand noblemen in a castellated abbey. The epidemic rages and kills the poor who were left outside to fend for themselves. Six months into their successful bid to avoid the contagion, the callous prince and his friends celebrate within the sealed confines of the abbey, when quite suddenly the disease invades their sanctuary and kills everyone.

## The Epidemiology

The story’s opening line, “The ‘Red Death’ had long devastated the country,” indicates that this is not a new disease outbreak but rather a known epidemic, ongoing or reemerging. High death rates leave Prospero’s dominions “half depopulated.” Poe gives a graphic description of the clinical features and outlines the course of the disease, from the earliest symptoms to the fatal outcome. The red death indiscriminately attacks all segments of the population, including healthy, immunocompetent hosts. Transmission, which seems to be accelerated by overcrowding, is from person-to-person contact and possibly aerosol inhalation. The death rate is highest in the abbey—all those confined inside die, while only half of those left outside do. Outside the abbey, once an infected person exhibits symptoms, others “shut him out from the aid and from the sympathy of his fellow-men.”

Exercising literary license, Poe confers upon the prince and his courtiers an extended reprieve of 6 months (after they isolate themselves from the general population) before the disease strikes them down and they die in the span of only half an hour. If a few of the noblemen came into the abbey already infected, the symptom-free 6 months could be explained by a long incubation period. Conversely, if the prince and his companions were initially uninfected, the microbe could have gained entry into the fortified sanctuary at a later time and caused the explosive epidemic. Even the strong physical barriers surrounding the abbey could not have stopped airborne or vector-borne transmission.

The geographic location of the story also offers clues about the epidemic. The presence of a prince and knights suggests a royal and feudal structure of governance somewhere in Europe. The use of “improvisatori” (from Italian *improvisatore*, an entertainer who improvises verse) (4) for courtly entertainment during their isolation suggests that the setting of this tale is Italy or a nearby European country.

## Possible Models for the Red Death

Even though a fictional product of Poe’s fertile and bizarre imagination, the red death is likely modeled after a disease within the author’s lifetime and experience. Some have speculated that Poe’s family history of tuberculosis (his mother, his adoptive mother, his wife, and possibly his brother died of the disease) may have prompted him to write about a similar disease in Life in Death—a story about a painter and his dying wife, who incidentally resembled Poe’s wife (5). Along the same lines, Poe’s experience of nursing his wife through her bouts of exsanguinating hemoptysis, cradling her head for hours, and wiping away the blood from her face may well have been on his mind when he mused about “the scarlet stains upon the face” of the afflicted in Masque of the Red Death.

As described by Poe, the red death seems to be some type of a viral hemorrhagic fever. Epidemics of yellow fever killed 100,000–150,000 in the United States from 1693 to 1905 (6). Northern ports (Boston, New York, Philadelphia, Baltimore), where Poe lived at various times in his life, were affected by yellow fever until 1822. He could have been inspired by a nationwide, severe epidemic of yellow fever in 1841 (a year before he wrote The Masque of Red Death), but yellow fever was a commonplace disease without any mystery attached to it. Like red death, yellow fever causes high body temperatures; body ache; damage to capillaries, which can result in bleeding from the nose and mouth; stools stained dark with blood; and (the most dreaded symptom) copious black vomit caused by gastric bleeding. Poe’s red death, however, has a much higher death rate and communicability. Besides, the eponymous jaundice of yellow fever is not described as a feature of red death. Poe named his fictional disease “red” death, probably to differentiate it from “black” death, otherwise known as the plague. “Red” death is also descriptive of the profuse bleeding characteristic of this fictional disease. Poe maximizes the horror of the disease by intentionally making it mysterious and universally fatal. By alluding to the black death, he invokes memories of the vast plague epidemics that ravaged the world.

## The Diagnosis

Poe’s description of the red death is in line with the clinical features of filovirus hemorrhagic fevers, which include Ebola and Marburg (7). Viral hemorrhagic fever outbreaks are rare but not unheard of in Europe. Crimean-Congo hemorrhagic fever and Marburg hemorrhagic fever have occurred in Europe. So, it is plausible for a filovirus to strike in Europe, which seems to be the setting of the red death. However, writing in 1840, Poe could not have known about Ebola or Marburg.

A potential, but unlikely, cause for the disease that Poe calls red death is some type of Marburg-like virus. During the self-imposed seclusion, Prospero provides his guests courtly entertainment in the form of buffoons, improvisatori, and ballet dancers. On these occasions, live animals were part of the program—we see them in the works of European artists who painted scenes from contemporary life. Peitro Longhi, a reputed Venetian artist, recorded the arrival of exotic animals at Carnevale in his painting Rhinoceros (in 1751) (8). The emblem of Carnevale was masked festivity, such as the one in Poe’s story. It is easy to conjure up monkeys imported from colonies in Africa to perform antics and entertain the princely court. The monkeys could have triggered a Marburg-like hemorrhagic fever—as seen at some European laboratories in recent times (9). However, this scenario requires the presence of African monkeys, which Poe does not mention. Besides, although this scenario could explain a contained outbreak in the abbey, it does not account for the major epidemic in the general population.

Filoviral diseases begin with abrupt onset of fever usually accompanied by myalgia. Poe’s description of “sharp pains” is eerily prescient. In the 1976 outbreak of Ebola in Sudan, knife-like sharp chest pain was an early symptom (10). Central nervous system involvement, also a feature of Ebola fever, may account for the “sudden dizziness” and delirium seen in red death. The prince and his courtiers chase the spectral imagery of the red death, and “gasp in unutterable horror at finding the grave-cerements and corpse-like mask which they handled with so violent a rudeness, untenanted by any tangible form.” These images of red death were evidently hallucinations. “Profuse bleeding at the pores” and “scarlet stains upon the body and face” of infected persons resemble the petechiae, ecchymoses, and mucous membrane hemorrhages found in half or more of Ebola fever patients.

 Like outbreaks of the red death, Ebola outbreaks remain shrouded in mystery, in spite of modern technologic investigations. Ebola’s origin and natural reservoir, as well as its location and behavior between outbreaks, remain poorly understood. Different strains of the virus have different death rates, with the Ebola- Zaire strain being the most lethal. Poe’s red death brings to mind some form of Ebola-like viral hemorrhagic fever of extremely high virulence.

Whether inspired by tuberculosis or yellow fever, the red death is clearly a concoction of Poe’s imagination. In honor of the creative genius that imagined Ebola fever long before the infection was recognized, the particular strain that causes red death might be named Ebola-Poe.
